# Malignant insulinoma with hepatic and pulmonary metastases associated with primary hyperparathyroidism. Case report and review of the literature

**Published:** 2008-04-15

**Authors:** Albu A., Zirnea A., Georgescu O., Terzea D., Jinga D., Fica S.

**Affiliations:** * Emergency University Elias Hospital, Bucharest, Endocrinology Department; ** „ Victor Babes” Institute, Bucharest; *** Emergency University Hospital Bucharest, Oncology Department; **** University of Medicine and Pharmacy „Carol Davila”, Endocrinology Department

## Abstract

Malignant insulinomas are rare tumors (10% of insulinomas) that often present as multicentric macro nodules with multiple liver metastases before diagnosis. We report the case of a 55 year old female with a medical history of severe hypoglycemic attacks for two months. Blood tests showed a decreased value of glycemia (30mg/dl) associated with increased insulin level (16μU/ml) and an increased glycemia/insulinemia ratio of 1.87 supporting the diagnosis of insulinoma. Abdominal CT showed a 1.5 cm mass localized in the head of the pancreas with disseminated hepatic tumors, confirmed as neuroendocrine metastases by biopsy (which proved the presence of a malignant insulinoma). Primary hyperparathyroidism was diagnosed based on mild elevation of calcium (10.4 mg/dl) associated with a high level of PTH (71,2 pg/ml). The coexistence of the two endocrinopathies suggested the presence of type 1 multiple endocrine neoplasia (MEN 1). Because of multiple hepatic masses and liver function impairment, surgery and hepatic artery embolization were not performed. Somatostatin analog therapy was started with symptomatic control in the beginning, but rapid loss of beneficial effect. Finally, systemic chemotherapy with doxorubicin was administered, but the disease was progressive and after three months we decided to stop it. The patient died at home after one month, probably in hypoglycemic coma.

Insulinomas are the most common islet cell tumors localized almost exclusively within the pancreas (over 99% of them and less than 1% in ectopic pancreas tissue) producing excessive amounts of insulin that can cause symptomatic hypoglycemia prompting patients to seek medical attention early during the course of their disease.

The Mayo Clinic published a 60-years study of insulinomas as rare tumors with an estimated incidence of 4 cases per million persons per year. There is a slight female preponderance, with 60% of new cases diagnosed in women and median age at presentation of 47 years (with a reported age range of 8 to 82 years) [**1**]. Most insulinomas are sporadic (90%), solitary (83-92%) and benign (90%), typically hypervascular tumors, measuring in 90% of cases less than 2 cm. Only 5.8-15% of them are multicentric macro adenomas (measuring over 2.5 cm), at a greater risk of harboring malignant lesions, with higher recurrence rates and unlike curative resection [**1**]. Epidemiological research regarding malignant insulinomas’ prevalence is not available, but the National Institute of Health (NIH; Bethesda, MD) reported 17 cases of malignant insulinomas treated between 1984-2004, as well as an additional of 45 patients identified in literature [**2**].

Approximately 50% of malignant insulinomas (half of them with disseminated hepatic metastases) and 7.6-16% of all insulinomas are associated with type 1 multiple endocrine neoplasia (MEN1). Insulinomas represent the second most common type presentation (20-45%) of MEN 1 after primary hyperparathyroidism (almost always present-95%), having an unfavorable outcome and higher recurrence rates [**3**].

Because of the rarity of the condition, we report the case of a 55 year old woman with simultaneous occurrence of primary hyperparathyroidism and metastatic insulinoma probably associated as MEN 1, with fulminant outcome (and severe prognosis).

## Case report

A 55 year old woman was referred to our Endocrinology Department in June 2006 for further investigation of multiple severe hypoglycemic attacks. She complained of sweating, tachycardia, palpitations, weakness, confusion and described episodes of hypoglycemic coma occurring in the last two months. She had severely limited physical activity because of hypoglycemia-related symptoms at minimal efforts. She had gradually gained weight (12 kg in the last two months) because of increased food intake to correct hypoglycemia.

The patient had been previously diagnosed and treated for high blood pressure.

Physical examination showed an overweight patient (BMI=29kg/m2). She had normal blood pressure (with antihypertensive treatment), normal pulse rate and body temperature. The abdomen was soft but with painless hepatomegaly. Physical examination of the cervical region revealed a mild goiter with normal consistency and no regional adenopathy.

Laboratory tests showed the presence of hypoglycemia (30mg/dl) associated with an increased value of plasma insulin (16µU/ml). Normal insulin level for glycemia under 45mg/dl must be below 6µU/ml. The calculated ratio of glycemia/insulinemia in our patient was 1.87 instead of the normal value of less than 0.25.

In a patient without previous treatment with insulin and sulfonylurea, failure to suppress endogenous insulin secretion in the presence of hypoglycemia is the hallmark of an insulinoma.

We also found elevated liver enzymes (ALT=47iu/l, AST=72iu/l, GGT=133iu/l). Decreased serum albumin (2.8g/dl) and prolonged prothrombin time indicated hepatic failure.

**Fig. 1:**
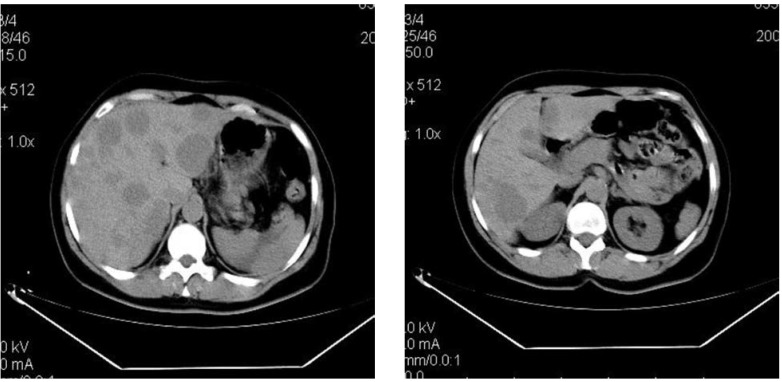
Abdominal CT ( june 2006) revealed a 1.5 cm mass localized at the head of pancreas and diseminated hepatic tumors

**Fig. 2:**
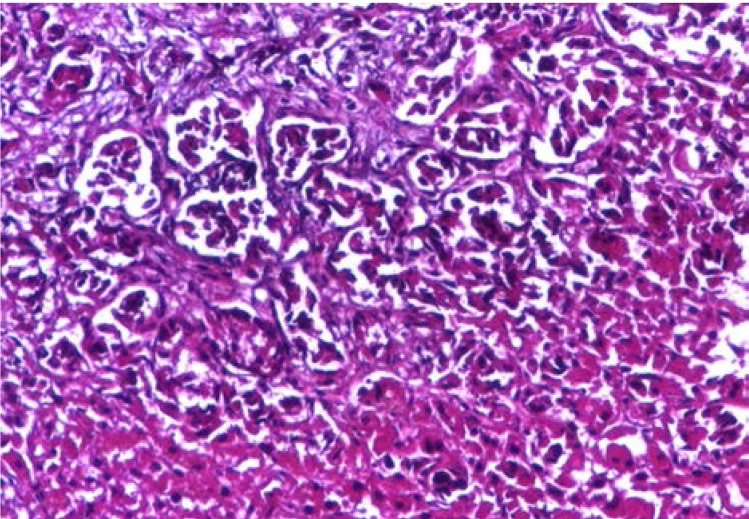
HE stain neuroendocrine carcinoma 100X

**Fig. 3:**
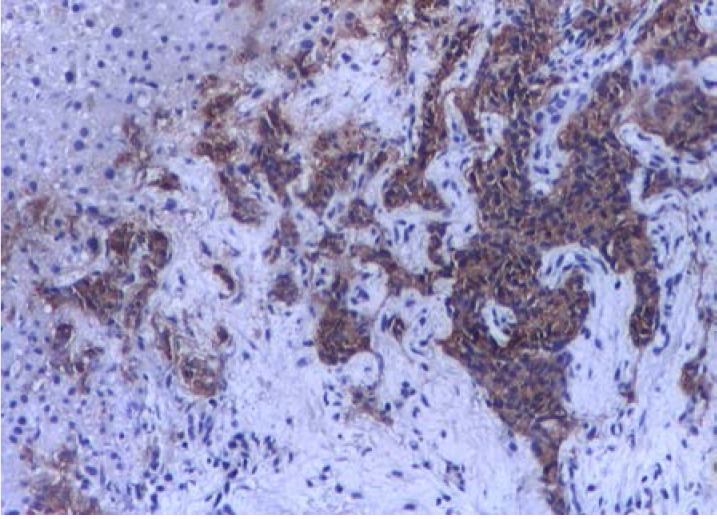
Neuroendocrine carcinoma cells positive for CD 57 IHC 100x

**Fig. 4:**
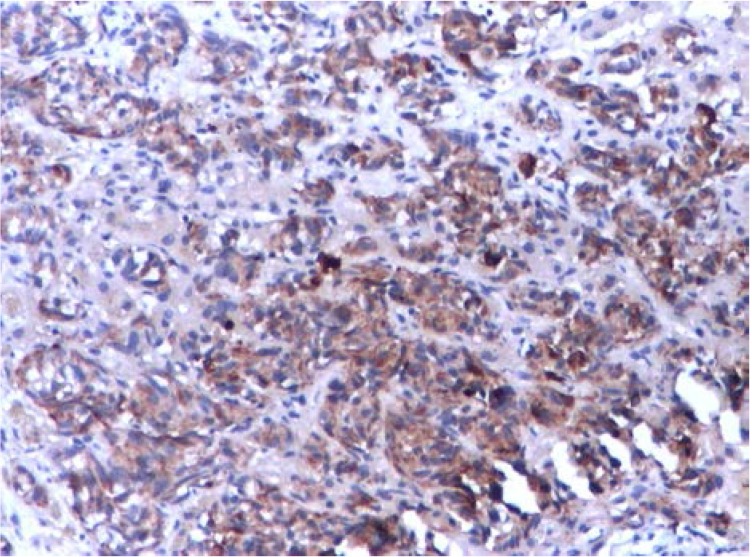
Neuroendocrine carcinoma tumoral cells positive for Chromogranin IHC 200X

**Fig. 5:**
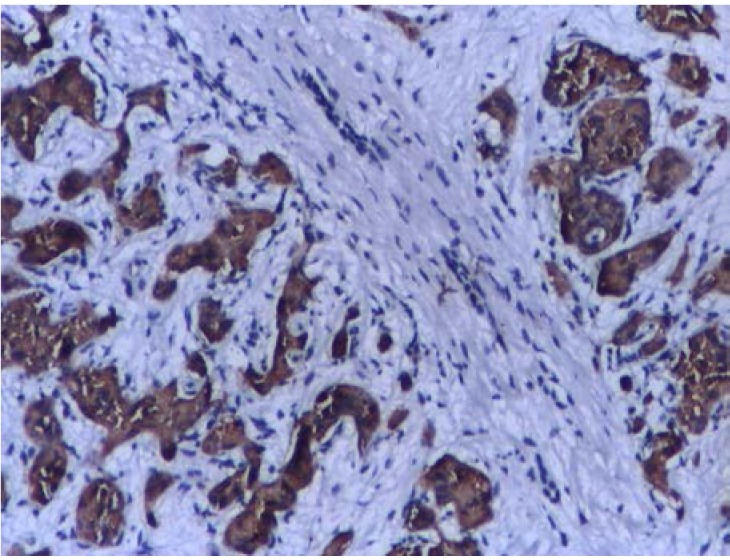
Neuroendocrine carcinoma tumoral cells positive for Synaptophysin IHC 100X

**Fig. 6:**
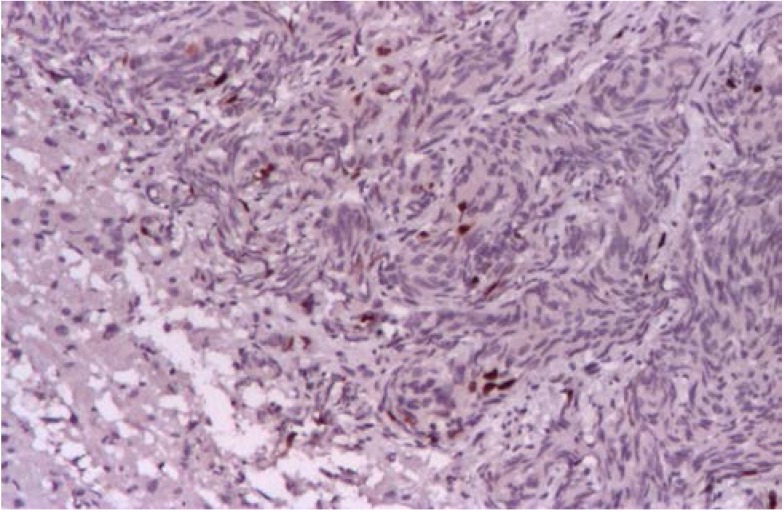
Imunostaining positive for Ki67 (~ 5%)

Abdominal CT revealed a 1.5cm mass localized at the head of pancreas and multiple tumors disseminated in both liver lobes (**Fig. 1**). Thoracic CT scan did not find any abnormal aspects suggestive of metastases. In order to establish the etiology of hepatic masses an ultrasound – guided liver biopsy was performed. Histological examination showed hepatic tissue with tumoral areas presenting an aspect of neuroendocrine carcinoma (**Fig. 2**). The immunohistochemistry study confirmed the neuroendocrine origin of the hepatic tumors (**Fig. 3**, **4**, **5**, **6**) showing tumoral cells with focal and diffuse expression for Chomogranin A, Synaptophysin and CD57; anti-Vimentin was positive in rare tumoral cells like Cytokeratin 18; anti-CD34 showed a denser vascular network; anti-Ki67 antibody displayed a nuclear expression in rare tumoral cells ( ~5%).

These findings established the diagnosis of malignant insulinoma with liver metastases.

Biochemical evaluation also revealed a mild elevation of total calcium 10.4mg/dl (normal upper range of serum calcium:10.2 mg/dl), normal serum phosphorus level of 4mg/dl (normal range: 2.5-4.5mg/dl) and increased alkaline phosphatase 405iu/l (normal range: 80-285iu/l).

In order to investigate the source of hypercalcemia, more tests were performed. We measured the PTH that revealed a mild elevated level of 71.2pg/ml (normal range: 10-69pg/ml) and 24 hour urinary calcium which was 180mg/dl (normal range between 100-300mg/24h).

Hypercalcemia with high PTH suggested the diagnosis of primary hyperparathyroidism.

Anterior neck ultrasound revealed a multinodular goiter with multiple nodules under 1cm, but did not give any information about parathyroid glands. A cervical CT was performed but it didn’t reveal any pathological parathyroid findings.

The coexistence of primary hyperparathyroidism and an enteropancreatic tumor (malignant insulinoma) suggested the presence of multiple endocrine neoplasia type 1 (MEN 1). Because of the high frequency of pituitary adenomas, especially prolactin-secreting ones among MEN 1 patients, we measured serum prolactin but we found a normal level: PRL=11.7ng/ml (normal ranges: 3-18 ng/ml).

In order to evaluate the presence of a pituitary adenoma we performed a cerebral CT scan which didn’t reveal any pathological aspects for the pituitary gland. We also considered the normal aspect of both adrenal glands on the abdominal CT in order to exclude an adrenal tumor, possibly associated with MEN 1. 

We started treatment and recommended a daily 250-300g of carbohydrate intake in eight meals (every 2-3 hours). In order to improve glycemic control, we administered verapamil 240 mg/day, but no result was obtained. 

The patient was referred to the surgical department but because of the magnitude of liver tumors and coexistence of liver dysfunction, surgical intervention was not an option. Impaired liver function was also a contraindication for hepatic artery embolization.

We decided to start medical treatment with a somatostatin analog. Our first option was long-acting lanreotide, 30mg at two weeks, but without response in terms of glycemic values. Instead, octreotide acetate 0.15mg/day (0.05 mg subcutaneously every 8 hours) was more effective, reducing both the frequency and severity of hypoglycemic episodes. We recommended that glucagon be available for the patient’s emergency use. 

Because of the general condition of the patient and the absence of advanced features of hyperparathyroidism, parathyroid surgery was not performed; we administered biphosphonate therapy as specific inhibitor agents of bone resorption (alendronate 70mg, once a week) associated with moderate dietary calcium restriction to 1000 mg/day and adequate hydration.

After two months (in August 2006) the symptoms were more difficult to control even with double doses of Octreotide (0.30mg/day subcutaneously).

Laboratory tests showed a worsening situation: glycemia=20mg/dl, insulinemia=190,7µU/ml; glycemia/insulinemia ratio=9,53, ALT=63iu/L, AST=117iu/L, GGT=272iu/L and a total level of calcium (11.1ng/dl).

**Fig. 7:**
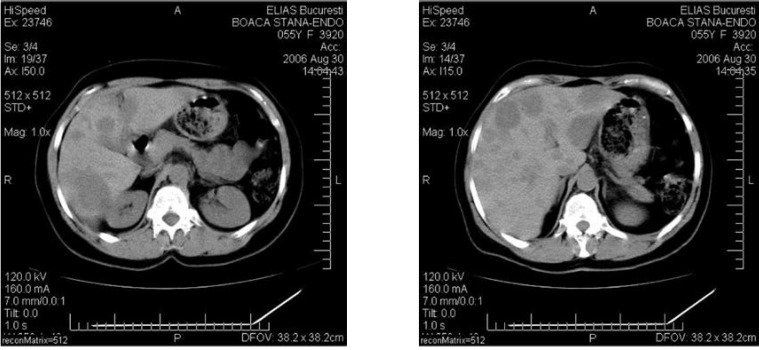
Abdominal CT revealed the progression of pancreatic tumor at 2,5 cm and the increase number and dimension of liver metastasis ( august 2006)

Abdominal CT revealed a progression of the pancreatic tumor (2.5cm) and increased number and dimension of liver metastases (**Fig. 7**).

We considered the disease as being progressive and a chemotherapeutical regimen with doxorubicin (100mg/day IV every 21 days) was instituted. After three months of chemotherapy no improvement was seen in biochemical or hormonal parameters. Moreover, CT exam showed a progression of hepatic masses and the presence of multiple sub pleural metastases. We decided to stop doxorubicin administration.

The patient died in December 2006 at home, probably in a hypoglycemic coma.

## Discussion

 Compared to other enteropancreatic tumors such as gastrinomas or glucagonomas, which are frequently malignant (46-60%), most of insulinomas are benign, only 5.8-15% being malignant or metastatic tumors [**4**]. Patients with malignant insulinomas represent a challenge for the managing clinician for diagnosis and therapeutic approach. Firstly, the presence of local invasion or metastases (most frequently in the peripancreatic lymph nodes and liver) should be established; secondly, the unregulated secretion of insulin and proinsulin-related products could lead to severe hypoglycemia and even hypoglycemic coma, which is a life-threatening condition. The average period of hypoglycemic episodes before diagnosis of insulinoma is established is generally 12-18 months [**2**]. In our case, the diagnosis of insulinoma was biochemical, confirmed after a 2 month period of symptomatic hypoglycemia due to the severity and high frequency of hypoglycemic symptoms.

Although most of neuroendocrine tumors are slow-growing and allow prolonged survival, some of them may show an aggressive behavior [**5**]. Therefore, identification of factors able to predict their natural history is important in order to approach appropriately a patient with insulinoma. Recently, WHO adopted a new classification of endocrine tumors of the pancreas based on clinical and pathological criteria such as: tumor size, local invasion, necrosis, structural atypia with prevalence of broad solid areas, cellular atypia with high nuclear cytoplasmic ratio, irregular distribution of chromatin, number of mitoses/10HPF, Ki 67 positive tumor cells, perineural and angioinvasion, tumor cells with immunoreactivity to neuroendocrine markers (synaptophysin, chromogranin A, insulin, proinsulin detected by immunohistochemistry), nuclear p53 protein accumulation [**6**, **7**]. According to this classification, enteropancreatic tumors may be well-differentiated endocrine tumors (benign or low-grade malignant), well-differentiated endocrine carcinoma and poorly-differentiated endocrine carcinoma. Ki 67 is an index of proliferative status, suggesting a more aggressive behavior when present in more than 2% of tumoral cells (**7**, **8**), as was the case of our patients. Still, the diagnosis of neuroendocrine carcinoma may be established only in the presence of metastases and/or invasiveness [**7**, **8**].

Despite these efforts to discover new histopathological criteria, there are no conclusive histological criteria or histochemical markers that reliably predict biological behavior. In an attempt to identify such predictors, genetic studies were performed to determine specific genetic changes associated with natural history and response to therapy.

Molecular genetic studies using conventional comparative genomic hybridization (CGH) have indicated chromosomal aberrations as initiating genetic events in malignant insulinoma pathology: multiple gene mutations, deletions of tumor suppressor genes, as well as simultaneous activation of proto-oncogenes. Chromosomal instability (CIN) is indicated to be a molecular marker for the metastatic potential of insulinomas. Activating point mutations were detected in several proto-oncogenes including codon 12 of K-ras, codon 61 of N-ras, c-myc, erb-2 [**9**]. Early suppression of apoptotic activity of c-myc is essential for survival of both neoplasic pancreatic β-cells and β-cells hyperplasia with high potential of vascular invasion [**9**].

Recently, micro array and quantitative real-time reverse transcription-PCR were applied to identify differential expressed genes between malignant and nonmalignant insulinomas as the most reliable indicators of malignant progression. CGH analysis revealed that the total number and types of chromosomal aberrations per tumor differs strongly between the benign and the malignant group (4.2 versus 14,1; p<0,0001) [**9**]. Chromosomal regions of maximum interest (CRI) simultaneously detected for a malignant tumor cell are: 11q24.1 (56%), 22q13.1 (67%), 22q13.31 (56%) and 9q32 (63%) [**9**]. Imunohistochemistry studies identified the expression of serpin peptidase inhibitor clade A member 1 (SERPIN1; alpha-1-antitrypsin) as up-regulated in malignant insulinomas (85.7% versus 14.3% of nonmalignant insulinomas). Serpin 1 may be considered as a marker of malignancy in insulinomas with clinical utility in recognizing patients more likely to develop an aggressive presentation [**10**].

In patients with neuroendocrine tumors we should always keep in mind the possibility of MEN, a group of heritable syndromes characterized by the presence of benign or malignant tumors in endocrine tissues. The presence of two MEN-related endocrinopathies suggests the diagnosis of MEN, with final confirmation only by genetic analysis. In our case, malignant insulinoma and primary hyperparathyroidism could be associated as MEN 1. MEN 1 represents a syndrome inherited as an autosomal dominant trait, the gene responsible for the disease being a tumor suppression gene called MENIN. Clinical entities described to be associated as MEN 1 are: hyperparathyroidism (95%), enteropancreatic tumors (30-80%), pituitary adenomas (most frequent prolactinoma, followed by growth hormone secreting tumors; 20-25%), carcinoid tumors (20%), adrenal adenomas (40%) and subcutaneous lipomas (30%) [**11**]. 

In patients with MEN 1 hyperparathyroidism is due to enlargement, possibly asymmetric, of multiple parathyroid glands. Although surgery is considered the treatment of choice of hyperparathyroidism in MEN 1, it represents an option that requires judgment in the presence of associated malignant enteropancreatic tumors. In such cases it may only be considered in nonprogressive enteropancreatic tumors. The presence of hyperplasic parathyroid glands in MEN 1 makes it necessary to perform an extensive exploration of four or more glands during initial surgery; therefore, a preoperative noninvasive imaging (ultrasonography or Tc99m-sestamibi, or both) is not imperative [**12**]. Surgery involves resection of three and a half glands, leaving half a gland in situ in an attempt to preserve residual parathyroid function and avoid hypoparathyroidism. Both the persistence and recurrence of hyperparathyroidism occur frequently in MEN 1 and probably reflect the high frequency of supernumerary glands and ectopically located parathyroid tissue [**13**].

We screened patients for other endocrine disorders usually related to MEN 1, but with no additional abnormal findings as shown by normal pituitary and adrenal CT scan, normal level of prolactin and clinical data.

Bearing in mind the hypercalcemia found in our patient, primary hyperparathyroidism clearly represents the main cause, but the possible contribution of PTHrP secreted by the neuroendocrine tumor or the osteolytic metastases can not be excluded.

Due to the relative rarity of malignant insulinomas, there is no consensus concerning the optimal management approach of such cases, but obviously it should be undertaken by a multidisciplinary team. Surgery represents the only curative option, although this is rarely possible [**5**]. It should be considered the treatment of choice when the general condition of the patient allows it, even in patients with hepatic metastases when at least 90% of tumoral tissue can be removed [**14**, **15**]; this is usually possible in the presence of solitary metastases or in those confined to one lobe. It was shown that in these cases the remission rate at three years is 50% and the survival rate is significantly higher (71% at five years) in comparison with those who receive palliative treatment [**16**]. Because of the slow growth rate of the majority of malignant enteropancreatic tumors and normal liver function despite gross tumor load [**17**], some authors recommend surgery with cytoreductive intent even in patients with less than 90% of tumoral tissue resectable, in order to improve symptoms and life quality [**14**]. There are not unequivocal evidences of prolonged survival in such cases. Moreover, patients with metastases at diagnosis and palliative treatment (54 of 64 cases reported) have a limited survival with an average period of two years [**1**, **2**].

For pancreatic tumors, surgeons perform either enucleation (for solitary tumors) or subtotal pancreatectomy (for multiple cephalic tumors) or distal pancreatectomy with spleen preservation. In selected cases with metastatic liver disease, hepatectomy could be followed by orthotopic liver transplant [**18**]. Several surgical procedures could be used in cases when curative surgery is not possible (radiofrequency ablation, cryosurgery with liquid nitrogen, ethanol injection), but their efficacy needs to be established by prospective clinical studies. Embolization or chemoembolization of hepatic artery is recommended in cases with progressives hepatic metastases in order to reduce tumor load and symptoms [**19**]; the presence of liver dysfunction represents a contraindication for this procedure.

Medical therapy is almost always required in patients with malignant insulinoma in addition to surgery (rarely curative) or in patients with inoperable disease. Somatostatin analogs and α-interferon could be used in patients with well-differentiated and slow-growing tumors; in poorly differentiated and progressive insulinomas chemotherapy represents an option [**5**].

Only 50% of insulinomas respond to treatment with somatostatin analogs, making it the least sensitive of all enteropancreatic tumors [**20**]. This is probably explained by the lack of type 2 somatostatin receptor in nonresponsive tumors. In such cases, diazoxide (not available in our country), verapamil or glucocorticoids should be considered for the control of symptoms. In cases responsive to somatostatin analogs, regular octreotide could be administered in a daily dose of 200-450mcg with symptomatic improvement and possibly tumor regression/stabilization [**20**, **21**, **22**]. There are studies suggesting that higher doses or long acting formulation of somatostatin analogs (octreotide, lanreotide) would provide better results [**20**, **21**, **24**, **25**]. Unfortunately, in time, somatostatin analog therapy ceases to be effective for the majority of patients. It was also suggested that patients resistant to lanreotide could be successfully treated with octreotide, explained by lack of cross-resistance between somatostatin analogs [**26**]. This was probably the explanation for good response to octreotide, but not lanreotide in our patient.

In our patient, the multidisciplinary team that managed the case weighed the risks and benefits of therapeutic alternatives. Because of the multiple liver metastases and general condition of patient, surgery was not an option. We tried a symptomatic treatment with somatostatin analog, octreotide, but the good initial results on blood glucose levels were rapidly lost, possibly due to disease progression or tachyphylaxis. The high cost of this therapy limited the administration of increasing doses of octreotide that could have probably achieved a better glycemic control and antiproliferative effects. Because of the progression of the disease, we chose to administer systemic chemotherapy with doxorubicin.

The natural history of malignant insulinoma depends on the extension of the disease, the associated comorbidities and biological behavior of the tumor. Malignant insulinoma tends to be fairly indolent and slow growing, although it does portend an unfavorable prognosis. Based on little differences between survival at 5 and 10 years, it was suggested that there are two subtypes of patients with hepatic metastases: 1) those with rapid progression of disease and death after an average duration of 6 months and 2) patients with slow progression and prolonged survival [**5**]. Mayo Clinic published a 60-year study of insulinoma and found a 15 year survival rate of 60-100% for localized disease, 40% for regional disease and only 29% for patients with distant metastases. Death may result from severe hypoglycemic reaction (coma) or spread of metastases in the whole body.

## Conclusions

 Although they are rare tumors, most of them benign, insulinomas should be regarded from the beginning as potentially malignant, with a major aggressive and fulminant outcome. Although the majority of malignant insulinomas progresses slowly, subsets of patients have an aggressive course of disease. Development of new criteria to predict biological behavior (mainly based on genetic markers) and adapted therapeutic algorithms are needed to optimize the outcome of this particular type of patients.

Our case is particular because the patient had an extensive disease at diagnosis with multiple liver metastases, with a fulminant and aggressive outcome leading to the death of the patient 6 months after diagnosis in the absence of surgical therapy.
